# Aeronutrient Therapy: A New Frontier in Systemic Drug Delivery

**DOI:** 10.3390/biomedicines13112788

**Published:** 2025-11-14

**Authors:** Stephen R. Robinson, Malav S. Trivedi, Flávia Fayet-Moore

**Affiliations:** 1 School of Health & Biological Sciences, Royal Melbourne Institute of Technology, Bundoora, VIC 3083, Australia; 2 Institute for Breathing and Sleep (IBAS), Austin Health, Heidelberg, VIC 3084, Australia; 3 Department of Pharmaceutical Sciences, Barry and Judy Silverman’s College of Pharmacy, Nova Southeastern University, 3200 South University Drive, Fort Lauderdale, FL 33328, USA; trivedi.mal@gmail.com; 4 FOODiQ Global, Sydney, NSW 2000, Australia; flavia@foodiq.global; 5 School of Environmental and Life Sciences, University of Newcastle, Ourimbah, NSW 2258, Australia

**Keywords:** inhalation, aerosol, vitamin, mineral, micronutrient, aeronutrient therapy

## Abstract

**Background:** Although the micronutrients (vitamins and trace minerals) essential for growth and normal physiological function are obtained from the diet, a substantial fraction of the human population is deficient in one or more micronutrients due to inadequate nutrition and/or malabsorption. **Methods:** This narrative review examines evidence that airborne micronutrients (‘aeronutrients’) are readily absorbed by the lungs, and preclinical and clinical evidence that inhaled iodine and vitamins A, B12 and D can enter the bloodstream. **Results:** Inhaled vitamin B12 resolves the symptoms and haematological features of pernicious anaemia with a bioavailability comparable to intramuscular injections and superior to oral formulations. Inhaled nebulised vitamin A restores serum levels in children with retinol deficiency. Randomised controlled trials have reported that inhalation of nebulised preparations of vitamins A, B12, magnesium and zinc are well tolerated and not associated with adverse health effects. Aeronutrient formulations have untapped potential for the therapeutic treatment of nutritional deficits, particularly in individuals with malabsorption or a low tolerance of injections. Aeronutrient therapy should be regarded as a medical intervention and be regulated accordingly, with efficacy and safety supported by scientific evidence, unlike the ‘vitamin vapes’ marketed by the wellness industry. **Conclusions:** Before this potential can be realised, a regulatory framework will need to be developed for aeronutrients. The high effectiveness of the pulmonary route introduces concerns regarding overdosing and toxicity which can best be addressed by categorising these formulations as prescription drugs that require regular monitoring of nutritional and health status.

## 1. Introduction

The advantages of inhalation for the uptake of nutrients and medicines have been recognised for millennia, with the oldest written records dating back 3500 years in Egypt, 3100 years in China and 2600 years in India [1,2]. However, scientific evidence for the pulmonary route being more effective for systemic drug delivery than the gastric route has only become apparent relatively recently.

Although the gastrointestinal tract is specialised for the absorption of nutrients from food, it is virtually impermeable to molecules larger than 600 Da [3]. Consequently, most proteins, polysaccharides and complex carbohydrates must first be degraded into smaller components (e.g., amino acids, simple sugars) before being absorbed. Furthermore, the extreme acidity of the stomach and abundance of proteases can compromise the stability and bioavailability of vitamins in the diet [4,5]. In contrast, while the human lung is specialised for the exchange of oxygen and carbon dioxide, it readily absorbs other airborne molecules into the bloodstream. Compared to the gut wall, the pulmonary alveoli have few proteases, a higher pH, fewer efflux transporters for xenobiotics and a lack of a thick mucus lining; features that permit the absorption of molecules as large as 160,000 Da [3].

Another difference between pulmonary and alimentary absorption is that molecules absorbed from the gut are transported by the portal venous system to the liver, where xenobiotics are removed from the circulation. However, molecules absorbed from the lungs into the bloodstream travel directly to the heart, bypassing the liver, and are then distributed throughout the body. Airborne hydrophobic molecules with a low molecular weight can be absorbed into the bloodstream much more rapidly than absorption via the gastrointestinal tract [3], which is why smoking is the favoured route of uptake for many of the drugs of addiction. Similarly, general anaesthetic agents used in surgery are primarily delivered by inhalation or intravenous injection, because unlike oral administration, these routes provide rapid induction of anaesthesia as well as superior control of the dosage and duration of action [6].

Since the pulmonary alveoli facilitate the absorption of airborne molecules, the present authors [7] postulated that airborne nutrients, or aeronutrients, such as trace minerals (e.g., zinc, manganese and iodine) and vitamins (e.g., vitamins A, B, D and E), might supplement dietary intake in instances where airborne concentrations are sufficiently high. We were the first to propose that naturally occurring airborne nutrients could be beneficial for health [7]. An important factor underlying this novel concept of aeronutrients is the massive differential between the volumes we consume and breathe. For instance, the recommended daily food intake to meet nutritional needs for adults [8] equates to 1.0–1.5 litres, which is a tiny fraction of the 18,240 litres of air that adults inhale each day, assuming 8 h of rest and 16 h of normal activity [9]. This difference means that the airborne concentrations of micronutrients can be five orders of magnitude lower than in food, yet still make a significant contribution to nutritional requirements [7].

Innumerable studies have shown that the delivery of pharmaceutical agents into the lungs via inhalers and nebulisers are highly effective methods for treating pulmonary diseases and infections (for reviews see [10,11]). Few studies, however, have investigated the use of inhalers and nebulisers to deliver aeronutrients into the bloodstream to treat systemic diseases and micronutrient deficiencies. The primary reason for this imbalance comes from research concerning the inhalation of trace minerals (e.g., manganese and copper) from air pollution and its negative effects on health. These studies have established that airborne trace minerals readily enter the bloodstream after inhalation, and that prolonged exposure to abnormally high concentrations (e.g., from industrial sources) can cause toxic levels of these minerals to accumulate in tissues such as the brain [12]. While concurring that exposure to air pollution ought to be minimised, we have noted that these data confirm inhalation as an effective route for absorbing trace minerals, and they support the possibility that aeronutrient therapy could treat micronutrient deficiencies [7].

**Figure 1:**
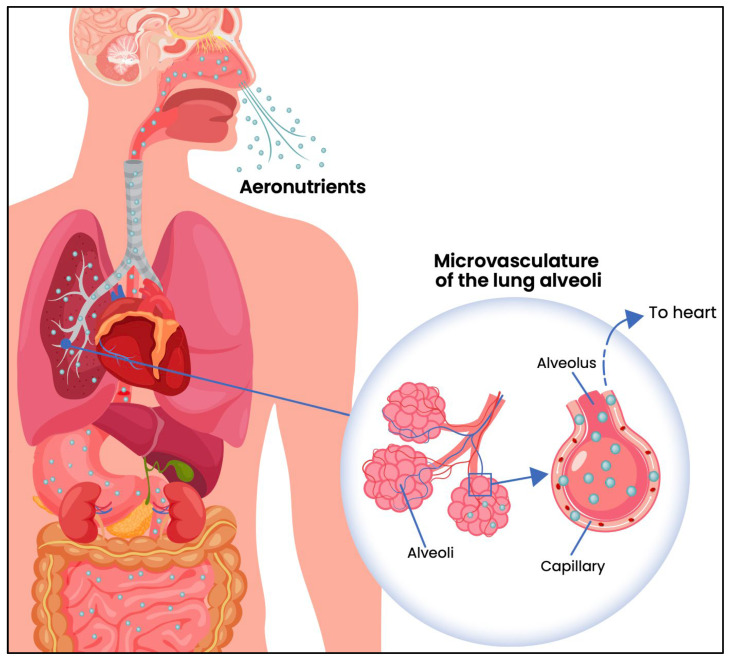
Diagram showing the diffusion of inhaled aeronutrients through the epithelial walls of alveoli, into the underlying capillaries and thence to the heart, bypassing the liver (redrawn from [7]).

The concept of aeronutrients needs to be differentiated from interventions that involve the inhalation of nutrients to treat diseases of the pulmonary epithelia. From a topological perspective, the pulmonary epithelia contribute to the external surface of the body, like skin cells and epithelia lining the gut wall. Animal studies have demonstrated that the inhalation of aerosolised micronutrients such as vitamin A, can be effective in treating lung injuries (e.g., [13,14]). Although such studies demonstrate that inhaled nutrients are generally well-tolerated by the pulmonary epithelia, they do not investigate the transfer of these nutrients across the epithelial wall into the bloodstream, whereas the present review focusses on airborne micronutrients that enter the circulation and have the capacity to benefit the internal organs (Figure 1).

The notion that aeronutrients can contribute to nutritional intake and status introduces the intriguing possibility that nutrient status can be intentionally supplemented by the inhalation of aerosols fortified with micronutrients. Here we: (i) review the supporting experimental and clinical evidence for the systemic delivery of aeronutrients; (ii) show how the commercialisation of inhaled micronutrients by the wellness industry is qualitatively different from aeronutrient therapy; and (iii) discuss the potential target markets and developmental considerations for aeronutrient therapy. The primary purpose of the present review is to highlight the unrealised potential of aeronutrients to treat nutrient deficiencies in humans, particularly in instances where oral supplementation is impractical or ineffective.

Literature searches for this narrative review were primarily based on queries of the Pubmed database (U.S. National Library of Medicine) using the terms ‘aerosol’, ‘aerosolised’, ‘nebulised’, ‘vaporised’, ‘vape’ or ‘inhaled’, in combination with ‘vitamin’, ‘mineral’, ‘nutrient’, ‘micronutrient’, ‘zinc’, ‘iodine’ or ‘magnesium’. No exclusion terms were applied, and no limits were placed on publication date.

## 2. Potential of Aeronutrients to Address Global Micronutrient Deficiencies

Two-thirds of the global population suffer from at least one micronutrient deficiency. A Harvard study in 185 countries found that 4–5 billion people have inadequate dietary intakes of vitamins A, C, D3 and E well as the essential minerals, iodine, magnesium and zinc [15]. Nutrient deficiencies are not limited to underdeveloped countries. In Australia and New Zealand for example, a review of national nutrition surveys and published literature revealed that of the 31 nutrients examined, the dietary intakes of 71% were inadequate. Micronutrient shortfalls were prevalent across the lifespan, and included all age groups and both sexes, with the highest prevalence of inadequate intake being for choline, potassium, calcium and vitamin B6 [16]. A similar investigation of dietary intakes in the U.S. [17] revealed strikingly similar findings, with 21 out of 24 nutrients (87.5%) being consumed at below recommended levels in at least one demographic group. Six micronutrients were consumed at inadequate levels across all demographic groups: vitamins D and E, calcium, choline, iodine magnesium and potassium.

Causes of micronutrient deficiencies are diverse and include an inadequate diet, insufficient exposure to sunlight, bowel malabsorption, increased nutrient needs during pregnancy, lactation or older age, dysphagia, and conditions that increase nutrient loss (e.g., consumption of diuretics, alcohol, or certain pharmaceuticals). Micronutrient deficiencies are associated with a wide range of chronic conditions and can increase susceptibility to infectious diseases [18]. For instance, a deficiency of vitamin A can cause blindness and increased susceptibility to measles and diarrhoea, while a deficiency of iodine causes developmental delays in children and goitre in adults [18].

While many cases of micronutrient deficiency can be treated with an adequate diet or the administration of oral supplements, the effectiveness of supplements can be limited by access (availability and cost), adherence (particularly in the longer term), and acceptability (taste, pill size). Further, conditions involving malabsorption or dysphagia are refractory to such treatments [19]. In these cases, regular intramuscular injections of micronutrients are the standard treatment. However, such injections can be painful, and can cause intramuscular haematomas or infections [20]. Consequently, they are not well tolerated by children, older individuals with sarcopenia or those with needle phobia. Needle phobia is surprisingly common; a recent survey of 2098 adults found that 63% had experienced needle phobia and half of the group had avoided medical treatment due to their fear of needles [21]. Injections of vitamins or essential minerals also suffer from the disadvantage that they must be administered by medical personnel, which increases their unitary cost, and introduces logistical issues, particularly in remote rural areas.

The delivery of micronutrients via aerosols offers an alternative that is painless, easy, and does not require administration by a medical professional. Research has shown that aerosolised vitamins can treat deficiencies without adverse side effects [7]. We suggest, therefore, that the therapeutic delivery of aeronutrients through metered doses may represent a new frontier in systemic drug delivery, helping to address global micronutrient deficiencies. The following sections review preclinical and clinical evidence that support the safety and efficacy of inhaled vitamins and trace minerals.

## 3. Experimental and Clinical Evidence for the Systemic Delivery of Aeronutrients

### 3.1. Vitamins

Schichlein and colleagues [22] reviewed literature that had investigated the potential of inhaled antioxidants to protect against pollution-induced lung injury. They concluded that preclinical and clinical studies have found no ill effects. Furthermore, several of the inhaled compounds, including vitamins C, D, and E, demonstrated antioxidant, antimicrobial, and anti-inflammatory benefits. This outcome indicates that the therapeutic inhalation of aerosolised vitamins could provide benefits without harming the lungs.

Aerosolised vitamin A: The active metabolite of vitamin A, all-trans-retinoic acid, is essential for phototransduction in the retina, and plays important roles in skin health and the prevention of cancer [23]. Chronic oral supplementation with retinoic acid derivatives is frequently prescribed for the treatment of skin diseases such as acne and for cancer prevention. However, oral supplementation can lead to skin reactions, teratogenesis, hyperlipidaemia and hepatic dysfunction. Brooks and colleagues used a rat model to investigate aerosolised vitamin A (all-trans-retinoic acid) as an alternative prophylactic treatment for patients at risk of developing secondary cancer [24]. They observed no ill effects and reported that plasma and liver levels of all-trans-retinoic acid increased after administration then remained stable for many hours. In contrast, intravenous all-trans-retinoic acid reached a much higher peak level and then declined to a lower level by 4 h. Brooks and colleagues commented that the lower stable levels achieved after inhalation, with a half-life of 5–17 h, are preferable as they are likely to cause fewer adverse effects.

Vitamin A deficiency is prevalent in countries where malnutrition and bowel infections are common. These conditions impair the absorption of vitamin A from the gut, reducing the effectiveness of oral supplements [25]. To examine whether aerosolised vitamin A could be beneficial in such cases, a placebo-controlled randomised supplementation trial was conducted, involving pre-school children in Ethiopia. In total, 25 children were treated with 2 mg of aerosolised retinyl palmitate (a form of vitamin A) every 2 weeks for 3 months, while a control group of 25 children received an aerosol that lacked retinyl palmitate [25]. After 3 months, serum retinol concentration had increased 2.1-fold and serum retinol-binding protein concentration had increased 1.8-fold in the treatment group (*p* < 0.001), whereas no changes were observed in the placebo group. Biesalski and colleagues concluded that aerosolised vitamin A appears to be a safe and effective route for boosting serum vitamin A levels in patients with malnutrition or malabsorption.

Aerosolised vitamin D3: 25-hydroxyvitamin D3 (vitamin D) is synthesised from 7-dehydrocholesterol when skin is exposed to sunlight, and it can also be obtained directly from a few food sources such as eggs, oily fish and UV-exposed mushrooms. Despite the abundance of sunlight, it is estimated that one in two people globally are deficient in vitamin D [26,27,28]. Vitamin D deficiency is associated with increased rates of cancer, inflammatory disorders, infectious diseases, type 2 diabetes, reduced bone density and cognitive dysfunction. The primary treatment is chronic oral supplementation with capsules of vitamin D. A preclinical study that investigated whether inhalation of vitamin D can restore levels in mice raised on a diet deficient in vitamin D found that exposure to low doses of nebulised vitamin D for 30 min per day for 7 days was sufficient to restore serum levels to normal [29]. In addition, a ceiling effect was observed, where higher doses did not raise serum levels further and longer treatment durations of 28 days continued to maintain serum vitamin D at physiological levels. No decrements in lung function were observed, leading the researchers to conclude that this therapeutic strategy is safe and effective.

Aerosolised vitamin B12: Vitamin B12 (cobalamin) deficiency is associated with pernicious anaemia, degeneration of the spinal cord, peripheral neuropathies, cognitive impairments [20], and elevated homocysteine, a risk factor for cardiovascular disease. While vitamin B12 deficiency can be due to an inadequate diet [15], it can also be caused by malabsorption, which due to physiological reasons, is exacerbated by old age. Vitamin B12 deficiency is present in 10–15% of persons aged over 65 and is brought about by a lack of intrinsic factor, decreased pepsin production and/or insufficient gastric acid that prevents the absorption of food-bound cobalamin [30]. Vitamin B12 deficiency due to malabsorption can also occur in younger persons who suffer from inflammatory bowel disorders. In such cases, oral supplementation is ineffective and regular intramuscular injections are needed. However, as noted in Section 2, these injections can be painful and are not well tolerated. This situation prompted an investigation of alternative methods of vitamin B12 delivery, particularly via the pulmonary route, as it is painless and can be self-administered. Table 1 summarises the primary observations from eight available studies that have investigated the efficacy of delivering vitamin B12 via the respiratory airways.

**Figure 2:**
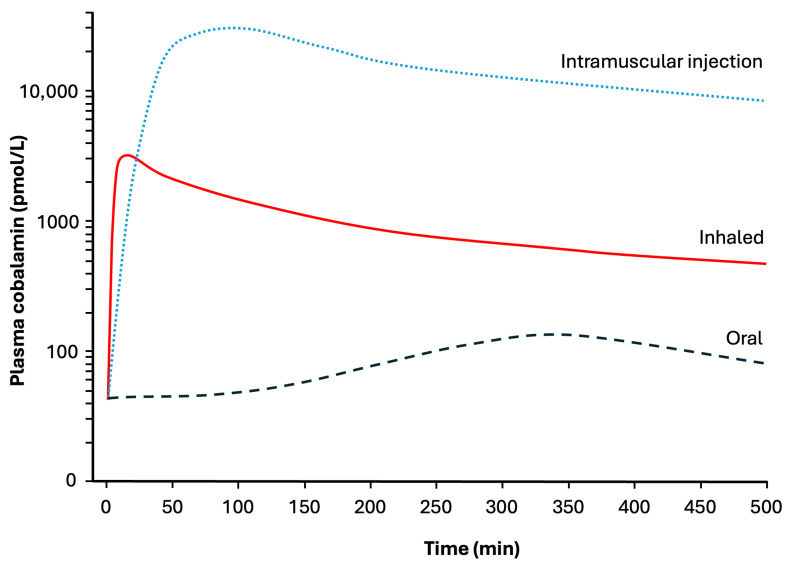
Schematic diagram comparing plasma concentrations of cobalamin (vitamin B12) in subjects with pernicious anaemia during the initial 8 h following administration of 1000 µg of cobalamin via three different routes. Based on data in [30,32,34,37].

While these studies are heterogeneous with regards to dosing and protocol, a consistent finding is that vitamin B12 administered via the respiratory airways is effective at restoring plasma vitamin B12 levels to the normal range and in normalising other haematological indices. Inhalation results in a more rapid spike in vitamin B12 levels than intramuscular injections or oral intake (Figure 2). The peak serum values attained via inhalation are approximately 10-fold higher than via the oral route, and 10-fold lower than by intramuscular injection (Figure 2). These differences in plasma concentrations are misleading, as most of the cobalamin is not bioavailable and will be eliminated in the urine. The relative bioavailability of vitamin B12 formulations has been estimated to be 2–6% for aerosolised preparations, 2–5% for intramuscular formulations and 2% for oral formulations [36]. With enough repeat doses, all three routes of administration are effective at treating pernicious anaemia, and the number of repeats required is inversely related to bioavailability.

Another consistent finding is that the inhalation of vitamin B12 is very well tolerated and results in few side effects. Five of the eight studies reported no adverse outcomes. Shinton and Singh [32] reported that following long-term therapy with inhaled cobalamin, one patient displayed a slight increase in linear markings in the lungs in a radiograph, indicating possible cobalt intoxication. In the study by Seth and colleagues [36], 14% of participants reported mild adverse events that resolved spontaneously without the need for treatment. There were no serious or severe adverse events, and global tolerability was rated as excellent. In a 3-month study of 60 elderly patients (mean age 78 years), 16.7% of participants reported mild adverse events, one patient died, and one was hospitalised. However, there was no control group, and it is unclear whether these events were treatment-related or due to old age [20].

### 3.2. Trace Minerals

Iodine deficiency is extremely common worldwide, due to the paucity of iodine in agricultural soils and the fact that the best sources of dietary iodine (seafood) are not widely consumed [15]. A remarkable body of research from Smyth and colleagues has suggested that in certain coastal regions of Ireland, air currents passing over dried kelp beds may pick up enough iodine to provide a significant fraction of the daily nutritional requirement [38]. Although aerosolised iodine has not yet been utilised to treat iodine deficiency, toxicity studies in humans using radiolabelled iodine have demonstrated that the absorption of iodine gas and submicron aerosolised iodine is highly efficient, with nearly 100% of the inhaled iodine remaining in the body after exhalation, where it is transported throughout the body and concentrated within the thyroid gland [39]. Such studies underscore the untapped potential of aerosolised iodine for the global management of iodine deficiency and insufficiency.

Magnesium is one of the most abundant minerals in the human body, where it is essential for muscle contraction, neurotransmission, DNA and protein synthesis, energy metabolism and as a co-factor for hundreds of enzymes [40]. Magnesium insufficiency is a common and global problem, due to an inadequate dietary intake and factors that commonly block its absorption from the gut [16,17,40]. Among its myriad functions, magnesium acts as a bronchodilator, and consequently magnesium sulphate has been used as an adjunctive treatment for acute severe refractory asthma, where it is administered intravenously or in nebulised form. A meta-analysis [41] compared 10 randomised controlled trials involving asthmatic children (*n* = 2301) who received nebulised magnesium sulphate as an adjunct treatment to salbutamol. While the nebulised magnesium had no effect on hospitalisation rates or asthma severity, it significantly improved peak expiratory flow rate and did not increase the frequency of adverse events when compared to salbutamol alone. Similarly, a meta-analysis [42] of 17 randomised controlled trials conducted in asthmatic adults, reported that nebulised magnesium sulphate significantly improved peak expiratory flow rate whereas intravenous magnesium sulphate had no effect. The nine trials involving nebulised magnesium sulphate (*n* = 1167) reported either no adverse effects or very minor adverse effects that did not result in withdrawal. These meta-analyses demonstrate that nebulised magnesium is well tolerated and safe, even in children and adults with severe breathing difficulties. Such findings provide confidence that nebulised magnesium could be an effective administration route for treating systemic magnesium deficiencies, particularly in cases where oral supplementation has been ineffective.

Zinc is an essential trace mineral that plays multiple physiological roles, including modulation of the anti-inflammatory response and defence from viral and bacterial infections [43]. Several clinical studies have examined whether the inhalation of aerosolised zinc salts can reduce the duration and efficacy of acute respiratory infections. For example, 80 volunteers with acute upper respiratory illness were provided with nasal spray bottles containing 0.12% zinc sulphate and instructed to administer two inhalations into each nostril four times daily until symptoms resolved, up to a maximum of 14 days [44]. Although no differences were observed in the duration of symptoms when compared to 80 controls who used an isotonic placebo, no adverse effects were recorded, indicating that aerosolised zinc is well tolerated. Despite the administration of nebulised zinc to increase zinc availability to the body and reduce symptoms, no measures of zinc biomarkers were reported. A meta-analysis [45] of 18 randomised controlled trials that used various delivery routes of zinc to treat upper respiratory infections, including 6 trials with a nasal spray (*n* = 1700) concluded that participants who received zinc intranasally were 1.8-fold more likely to recover before those who received the placebo. Furthermore, the percentage of adverse events associated with intranasal administration did not differ from the placebo condition and was less than that seen with other routes of administration. These outcomes support our proposal that aerosolised formulations of zinc may represent a safe route for treating systemic zinc deficiencies.

A summary of the experimental and clinical evidence for aeronutrients discussed, and the gaps and opportunities for research are summarised in Table 2.

## 4. Target Markets and Developmental Considerations for Aeronutrient Therapy

The physiology of aeronutrient absorption along with the research findings reviewed above, indicate that aeronutrient therapy may have utility for the treatment of systemic nutrient deficiencies and insufficiencies, particularly in instances where the oral route is contraindicated or ineffective. Eight small clinical studies have confirmed the benefits, tolerability and safety of inhaled vitamin B12. While we expect the aerosolised delivery of vitamin A, vitamin D, iodine, magnesium and zinc to be equally efficacious, their effectiveness is currently supported by a smaller body of evidence. As far as we are aware, no preclinical or clinical research has been conducted into the aerosolised delivery of any other micronutrients.

Precedents, such as the use of oxygen therapy to treat chronic obstructive lung disease and inhaled insulin for diabetes care, demonstrate that under the right conditions, inhalation can be a safe and effective delivery system. Aeronutrient therapy offers the potential for a fast and effective route to improve nutritional status in humans, particularly amongst those at greatest risk of suboptimal intake. Despite the potential to improve human health, many challenges need to be overcome before aeronutrient delivery can translate into clinical outcomes.

The optimal target groups for aeronutrient therapy are those where conventional supplementation routes are either impractical or ineffective. Older adults have several risk factors that contribute to suboptimal oral micronutrient intake, including dysphagia, cognitive decline, and reduced gastrointestinal absorption, as well as a higher need for certain micronutrients (e.g., vitamin D). Vitamin B12 deficiency in vegans, or persons with pernicious anaemia, are also potential target groups. Aeronutrient therapy may be a better alternative for infants, patients with needle phobia, persons with bleeding disorders or those with poor healthcare access. Other potential groups include those who are deficient, have malabsorption (e.g., post-bariatric surgery, inflammatory bowel disorders), dysphagia, achlorhydria, or have poor oral adherence. Aeronutrient therapy may also represent an acceptable option for long-term maintenance of nutritional status after bolus injections (e.g., vitamin D or vitamin B12) in instances where diet is inadequate (e.g., vegan diet). Finally, there are opportunities for applications that are targeted to extreme environments such as spaceflight, military or disaster relief, where oral intake of micronutrients may not always be suitable or accessible.

## 5. Commercialization of Inhaled Micronutrients by the Wellness Industry

Between 2006 and 2010, e-cigarettes became widely available and were marketed as a safer alternative to smoking tobacco. E-cigarettes or ‘vapes’ use a lithium battery to raise a heating coil to temperatures of up to 300 °C so that a liquid formulation (consisting of flavourings, nicotine and propylene glycol) can be vaporised into a flavoured aerosol that can be inhaled into the lungs. It is now recognised that while vapes are less harmful than tobacco cigarettes, chronic use is associated with an increased incidence of cardiovascular and respiratory diseases, periodontal disease and mental health issues [46]. The toxicity of vapes has been attributed to various contaminants such as tin, lead and nickel, as well as additives such as nicotine, tetrahydrocannabinol and vitamin E acetate [46,47]. The latter is added as a thickening agent, yet when subjected to the high temperatures in vapes, pyrolysis of vitamin E acetate produces toxic ketene gas, carcinogenic alkenes and benzene [47]. It should be noted that vitamin E acetate has a different molecular structure to vitamin E, which is harmless to pulmonary cells and appears to have anti-inflammatory properties in animal studies [48].

Shortly after 2010, ‘vitamin vapes’ appeared on the market. They were touted as a healthier alternative to e-cigarettes because they were nicotine-free and contained vitamins, amino acids, and other bioactive agents such as caffeine or melatonin. Unlike the inhalable pharmaceutical products salbutamol [49] and insulin [50], most of these vitamin vapes were focussed on the self-prescribed consumer wellness sector. *NutriAir* (NutriAir NV, Nutrition, LLC, Clearwater, FL, USA) was launched in 2013 in Florida as a direct-to-consumer brand of refillable vitamin inhalers, offering proprietary blends branded as ‘Focus’ and ‘Immune’. These products were marketed as fast-acting and ultra-micronised preparations for rapid absorption, underpinned by ‘micro-aerosolisation’ principles. In 2014, *NutroVape* (Nutrovape, LLC, St. Petersburg, FL, USA) introduced disposable vitamin inhalers for energy (containing caffeine and taurine), relaxation (containing melatonin or chamomile), or to ‘feel-good’ (containing vitamins B12 and C). Their inhaler could deliver 5 mg of active ingredients per inhalation with up to 200 doses per stick. *Sparq Life*, *Inc.* (New York, NY, USA), established in 2015 in New York, markets a refillable inhaler that is 100% recyclable and laboratory-tested, providing vitamins C, D, and B-complex, alongside amino acids (L-theanine) and herbal extracts (e.g., green tea, blueberry). *Breathe™* (Breathe B12, Minneapolis, MN, USA) has been more focussed in its approach and only offers vitamin B12 inhalers that are marketed as ‘supplement diffusers’ or ‘B12 bars’ in a variety of flavours and product forms. Breathe™ claims their inhaler is a safe, clean-room-manufactured, nicotine-free, sugar-free alternative to smoking.

It is unclear which vitamin vapes contain vitamin E acetate or whether any of the nutraceuticals that are added to these vapes undergo a toxic transformation when exposed to high temperatures. Furthermore, because vitamin vapes are primarily marketed to the wellness sector, they have avoided regulation by federal health authorities, and it has not been necessary to characterise the pharmacokinetic profiles of these vapes. At present, there are no published data regarding the health benefits or adverse effects of vitamin vapes.

Although vitamin vapes have been commercially available for the past 15 years, they differ from the concept of aeronutrient therapy in almost every respect, including their formulation, regulation and availability. These differences are summarised in Table 3 and are reviewed in the following sections.

## 6. Regulatory Considerations

Vitamin vapes have largely avoided regulation by being marketed as wellness products, nonetheless, company websites and product packaging occasionally include unsubstantiated health claims. For instance, in 2021, the United States Food and Drug Administration [51] issued a warning letter to the parent company of NutroVape for regulatory violation due to their unapproved claims of a benefit for health, including for treatment of hangovers and improvement of sleep. The FDA noted that such claims established NutroVape products as unapproved new drugs. The parent company was requested to withdraw all health marketing claims for its products within 15 days. The FDA also issued a broader advisory to all manufacturers of vaping products that contain vitamins [52], noting that “*these products are being illegally sold with unproven claims and could be harmful if used…the FDA may take enforcement actions to prevent the products from reaching consumers*.” It is unclear whether the FDA intends to enforce compliance, as inhaled vitamin products are still being marketed with claims of health benefits, despite the FDA having not issued a close-out letter to indicate that the violations and concerns have been addressed.

Unlike vitamin vapes that are marketed to health-conscious consumers, the purpose of aeronutrient therapies is to treat micronutrient deficiencies and insufficiencies. This difference means that aeronutrient preparations will be automatically scheduled as therapeutic drugs and will have to meet federal guidelines, such as the Therapeutic Goods Administration’s (TGA) guidelines in Australia. These guidelines require documented evidence that a manufactured product has a consistent quality and is safe for human consumption. Particle size, excipient toxicity, and aerosol device dose reproducibility must meet pharmaceutical standards for pulmonary drug delivery [53], emphasizing the need for careful testing and selection of pharmaceutical-grade excipients. In view of the adverse health outcomes associated with e-cigarettes, it will be important to conduct pharmacokinetic studies and to demonstrate that aeronutrient therapy formulations are safe for long-term use.

An unexpected obstacle to the approval of commercial aeronutrient formulations lies in the wording of the Federal Food, Drugs and Cosmetics Act, which defines the term ‘dietary supplement’ as a product intended for ingestion [51]. Since aeronutrient products are not intended for ingestion, they do not meet the definition of a dietary supplement under the Act. Thus, before aeronutrient therapy can be used to treat micronutrient deficiencies, it will first be necessary to amend the Act and consequently, the regulatory framework, to recognise that nutritional supplements can also be absorbed via inhalation.

### Safety and Efficacy

Given the substantial scientific evidence for their rapid and safe uptake in humans, including for vitamin B12 and iodine, the most obvious challenge is to establish the safe upper limits for aeronutrient therapy. The delicate lung environment poses some risks that need to be investigated, particularly if aeronutrient preparations are provided in nanoparticle form [54]. For aeronutrient delivery systems, these insights are crucial for designing safe and effective formulations that utilise lung absorption pathways while minimising harmful outcomes. Short-term safety trials are required to monitor for irritation, allergies, and acute side effects, while long-term studies are necessary to determine the effects on lung and organ physiology as well as whether they demonstrate cumulative toxicity.

Research is needed to inform the design of aeronutrient delivery systems that target alveoli for optimal absorption. The formulation needs to consider the size, charge and solubility of an aeronutrient, to balance absorption with safety. Pharmacokinetic studies are needed for each inhaled micronutrient to determine the amount absorbed into circulation and to compare this with the absorption rates of oral and injectable forms [3], as well as the proportion absorbed via the alveoli, the bioavailability and the amounts exhaled or excreted. To optimise absorption, research will be required on the types of formulations for delivery, ranging from nanoparticles to nano-encapsulated aerosols or, like their oral counterparts, time-released particles. Researchers may be able to rely in part, on previously validated inhalation testing as well as the bioequivalence guidelines for regulatory and clinical approval [55]. New devices for delivery may need to be developed, likely leveraging existing devices like those for asthma medications that provide strictly metered dosages. Finally, efficacy trials are needed that compare inhaled versus oral and injectable forms for increasing nutritional status, and to determine whether aeronutrient therapy can address the shortfalls seen with other routine routes of micronutrient administration.

Unlike oral supplementation, where dose is tightly controlled, inhalation bypasses first-pass metabolism, and uptake into the bloodstream is direct and rapid (Figure 1). The inhaled dose required for aeronutrient therapy is likely to be significantly lower than that for oral ingestion, and this difference necessitates greater caution. The challenge lies in ensuring uniform delivery per breath. Although the availability of oral micronutrients is unrestricted and unregulated, we anticipate that aeronutrient therapy will need to be restricted to vulnerable groups, and the dosing regimen tailored to individual needs. Oral nutritional supplements are poorly regulated globally [56]; their low cost and easy access enables consumers to self-prescribe mega-doses of vitamins and trace minerals without being able to screen themselves for signs of toxicity [57]. For example, the TGA in Australia restricted over-the-counter sales of vitamin B6 because excessive doses from multiple supplements had resulted in several cases of neuropathy [58]. If oral vitamin supplements can be toxic, then greater care needs to be taken when directly delivering these vitamins to the lungs. Restricting access by prescription to vulnerable or clinically indicated groups is likely to be the safer and more evidence-based option. There will be a need for ongoing monitoring of health and micronutrient status to ensure that aeronutrient therapies remain safe and effective.

## 7. Limitations and Future Directions

This review has drawn attention to the unexplored potential of aeronutrients to treat micronutrient deficiencies in humans. A limited number of preclinical and clinical studies have shown that inhaled iodine and vitamins A, B12 and D can enter the bloodstream and reverse micronutrient deficiencies. Inhaled micronutrients appear to be well-tolerated and have few adverse outcomes, at least in the short-term. Although these findings are promising, the current body of data is small, and further research is required before aeronutrient therapy can be adopted as a mainstream approach to address the growing micronutrient inadequacies globally.

The primary limitations that need to be addressed include:

(i) The lack of long-term safety data;
(ii) The need for direct comparisons of the efficacy, safety, compliance and cost of the different routes of micronutrient administration (oral, intramuscular and inhaled);
(iii) The need for more preclinical and clinical studies of aeronutrients, particularly with regards to individual micronutrients that have not yet been investigated;
(iv) The development of delivery systems that can reliably provide metered doses of specific micronutrients to individuals for whom oral or intramuscular interventions are unsuitable.

Since more than half of the world’s population suffers from micronutrient deficiencies, new approaches to address this problem are urgently needed. The field of aeronutrient therapy is at an embryonic stage of development, and it is likely to take a decade or more before this approach can be employed at scale. Nonetheless, the evidence presented shows early promise as an adjunct to existing therapies and it may prove to be more efficacious and cost-effective in circumstances where conventional therapies are impractical or have limited effectiveness.

## 8. Conclusions

Realisation of the full therapeutic potential of aeronutrients will require interdisciplinary collaborations across nutritional science, pulmonary medicine, pharmaceutical engineering and regulatory policy. From determining the appropriate dosing targets and key deficiencies of public health significance to assessing the risks of chronic inhalation, to particle design and nebuliser technologies, the collaboration and frameworks required will resemble those that led to the availability of inhaled insulin.

The regulatory framework for safety and efficacy monitoring could include a requirement that aeronutrient therapies are dispensed only through pharmacies under a physician’s prescription. This will enable clinicians to track serum titres of the specific micronutrients before and after treatment, building clinical evidence, and monitoring efficacy while reducing the risk of overdosing. Limiting access to prescription-only use will make it clear to the public that aeronutrients have a medical benefit and are distinct from unregulated wellness products. Further, as current nutritional supplement regulations only recognise oral and injected nutrients, aeronutrient delivery will likely require a new classification. In turn, clinical guidelines will be needed to specify who can prescribe and the protocols for monitoring efficacy and risk.

Finally, alongside regulatory frameworks, public education and communication are essential. For decades, inhalation has been linked to health risk (smoking, vaping) or to disease management (oxygen therapy, asthma inhalers). This negative association needs to be shifted towards the more positive view that inhaling nutrients is as health-giving as breathing fresh air. Aeronutrient therapy is an unexplored field that offers new ways to address global micronutrient deficiencies and improve health outcomes globally. Realising this vision will depend on building the scientific evidence, establishing a precise regulatory framework, and educating health professionals about the considerable potential of this emerging therapeutic approach.

## Figures and Tables

**Table 1:** Primary observations of studies in humans that have used inhaled or intranasal sprays of vitamin B12.

Subjects	Treatment Duration	Primary Observations
3 adults with pernicious anaemia [31]	3–4 months	Resolution of clinical and haematological symptoms.
18 adults with pernicious anaemia; 3 adults with vitamin B12 deficiency [32]	5–15 months	Complete clinical and haematological remission within 4–8 weeks.
6 adults with vitamin B12 deficiency [33]	5 weeks	8-fold spike in serum levels after 1 h; 3-fold increase after 4 weeks. Serum levels remain high 1 week after administration.
10 healthy adults, crossover dose study [34]	4 h	Dose-dependent increases in plasma cobalamin, peaking after 28–35 min at 7–14-fold of baseline, declining slightly thereafter.
10 senior adults with vitamin B12 deficiency. Single intramuscular or intranasal dose [30]	48 h	After an intranasal dose, plasma cobalamin peaked at 42 min at 5–10-fold baseline then declined over 48 h. After intramuscular injection, plasma cobalamin peaked at 5.4 h and declined slowly over next 42 h, the peak was 38-fold higher than after intranasal administration.
10 children with vitamin B12 deficiency [35]	47–266 days	A 15-fold increase from baseline in plasma vitamin B12. Deficiency was resolved in all cases.
81 adults with vitamin B12 deficiency [36]	6 weeks	Normal plasma cobalamin levels were attained after 2 weeks in all participants; haemoglobin and reticulocyte levels were normal after 6 weeks in all participants.
60 senior adults with vitamin B12 deficiency [20]	90 days	Normal plasma cobalamin levels were attained after 1–4 weeks in all participants and remained stable thereafter.

**Table 2:** Summary of reviewed preclinical and clinical evidence supporting the safety and efficacy of aeronutrient therapy for the treatment of systemic deficiencies of vitamins and essential minerals.

	Vitamin A	Vitamin B12	Vitamin D	Iodine	Magnesium	Zinc
**Administration route(s)**	Inhaled nebulised aerosols	Inhaled nebulised aerosols; intranasal sprays	Inhaled nebulised aerosols	Inhaled gas or aerosol	Inhaled nebulised aerosols of magnesium sulphate	Intranasal sprays of zinc sulphate or zinc gluconate
**Clinical trials**	Ethiopian preschool RCT improved serum retinol	8 clinical studies, rapid plasma increase, correction of deficiency comparable to I.M.	N.A.	Pulmonary absorption of inhaled radiolabelled iodine is near 100%	10 RCTs in asthmatic children; 9 RCTs in asthmatic adults. Acute administration. Improved pulmonary function	6 RCTs of adults with common cold. 1.8-fold more likely to recover before placebo
**Preclinical research**	Rats. Serum retinoic acid increased	N.A.	Deficient mice. Serum levels restored to normal	Airborne iodine in coastal areas is associated with increased urinary iodine	N.A.	N.A.
**Potential risks of overdose**	Teratogenic and hepatotoxic	Cobalt intoxication; generally safe in high dose	Persons with kidney disease. Fatigue, muscle weakness, kidney damage	Thyroid dysfunction	Persons with kidney disease. Nausea, muscle weakness, hypotension	Nausea, copper-deficiency; generally safe in high dose
**Adverse effects**	None reported	Excellent tolerability; a few mild adverse events	None reported	None reported	None reported or clinically insignificant effects	Not different from placebo
**Gaps and priority areas for research**	Pharmokinetic and long-term safety trials. Efficacy in malnourished groups	Long-term RCTs. Delivery device optimisation	Pharmokinetic and long-term safety trials. Efficacy in malnourished groups	Pharmokinetic and long-term safety trials. Efficacy in malnourished groups	Pharmokinetic and long-term safety trials. Efficacy in malnourished groups	Pharmokinetic and long-term safety trials. Efficacy in malnourished groups

**Abbreviations:** I.M. intramuscular injection; N.A. = not applicable; RCT = randomised controlled trial.

**Table 3:** Comparison of the formulation, regulation and availability of vitamin vapes to that envisaged/recommended for aeronutrient therapies.

	Vitamin Vapes	Aeronutrient Therapies (Recommendation)
Formulation	Flavouring agents, propylene glycol, several nutraceuticals, stabilisers, thickening agents.	A single vitamin or essential mineral, antioxidants and a carrier solution.
Mode of delivery	High temperature vaporisation via an e-cigarette device.	Room temperature mist via a metered dose inhaler or dry powder inhaler.
Dosage	Unregulated daily intake determined by the user.	Recommended by physician.
Availability	Unregulated purchase via internet.	Via prescription.
Target group	Health-conscious young consumers.	Patients with micronutrient deficiencies that are refractive to conventional treatments.
Safety monitoring	None required.	Monitoring of nutritional status and health via blood tests and health checks.
Quality control	None required.	Particle size, excipient toxicity and aerosol device dose reproducibility must meet pharmaceutical standards for pulmonary drug delivery.
Pharmacokinetics	None required.	Studies conducted to determine the amount absorbed into circulation via the alveoli, the bioavailability, and amounts exhaled or excreted.
Toxicity control	None required.	Short-term safety trials to monitor for irritation, allergies, and acute side effects. Long-term studies to determine the effects on lung and organ physiology and cumulative toxicity.
Medical regulatory controls	None required.	Must conform to all federal and country guidelines pertaining to therapeutic products.
Packaging	Must avoid making health claims.	Must list all ingredients, dosage, indicate expiry date, and include an insert that lists known contra-indications, side-effects, etc.

## Data Availability

Not applicable.

## Abbreviations

The following abbreviations are used in this manuscript:

FDA	U.S. Food and Drug Administration
TGA	Therapeutic Goods Administration (Australia)
